# Predicting the risk of metabolic-associated fatty liver disease in the elderly population in China: construction and evaluation of interpretable machine learning models

**DOI:** 10.3389/fmed.2025.1678076

**Published:** 2025-10-20

**Authors:** Yingxin Zeng, Chaobing Yang, Xin Yang, Xinmei Zhang, Guodong Xia

**Affiliations:** ^1^Department of Gastroenterology, The Affiliated Hospital of Southwest Medical University, Luzhou Sichuan, China; ^2^Department of Critical Care Medicine, The Affiliated Hospital of Southwest Medical University, Luzhou Sichuan, China; ^3^Health Management Center, The Affiliated Hospital of Southwest Medical University, Luzhou, Sichuan, China

**Keywords:** machine learning, metabolic-associated fatty liver disease, predictive model, older adults, random forest

## Abstract

**Introduction:**

With the rising incidence of metabolic dysfunction-associated fatty liver disease (MAFLD) in the elderly population, this study aimed to develop an optimal screening model by comparing ten different machine learning (ML) algorithms to identify high-risk elderly individuals using routine health examination data.

**Methods:**

The study included 2,635 individuals aged 60 years and older who underwent annual health examinations at the Health Management Center of Southwest Medical University Affiliated Hospital from January to December 2024. Initial feature selection was performed using the least absolute shrinkage and selection operator (LASSO) regression, followed by univariate and multivariate logistic regression analysis to identify nine independent predictive factors. Predictive models were constructed using 10 ML algorithms, and model performance was evaluated based on discriminative ability, calibration ability, and clinical utility. Feature importance was visualized and individual-level interpretability was provided using the Shapley Additive exPlanations (SHAP) method.

**Results:**

The final analysis included nine variables. After 10-fold cross-validation and hyperparameter tuning, the Random Forest (RF) model performed best, achieving an area under the curve (AUC) of 0.892 (95% CI: 0.870–0.914) in the validation cohort. Feature importance analysis revealed that the TyG-BMI index, height, and albumin levels played significant roles in predicting MAFLD risk.

**Discussion:**

Machine learning models, particularly the random forest algorithm, can effectively predict the risk of MAFLD in the elderly population. These models may assist clinicians in early screening and intervention, thereby improving patient outcomes.

## Introduction

Metabolic-associated fatty liver disease (MAFLD), formerly known as non-alcoholic fatty liver disease (NAFLD), is a condition strongly associated with metabolic dysfunction, including obesity, type 2 diabetes mellitus, insulin resistance, and metabolic syndrome (1). With the global acceleration of population aging, the prevalence of MAFLD is rising among older adults (2).

Metabolic-associated fatty liver disease not only impairs liver function but is also closely linked to a range of extrahepatic complications. Studies have shown that MAFLD significantly increases the risk of both fatal and non-fatal cardiovascular events, and patients with MAFLD are more likely to develop chronic kidney disease and type 2 diabetes compared to healthy individuals (3–5). Moreover, MAFLD can progress to non-alcoholic steatohepatitis (NASH), liver fibrosis, cirrhosis, or even hepatocellular carcinoma (HCC), posing a serious threat to patients’ health and survival (6). These complications are more prevalent in the elderly, further exacerbating the disease burden. Therefore, early identification of MAFLD in older adults is crucial for reducing healthcare costs, improving prognosis, and enhancing quality of life.

Abdominal ultrasonography is a widely used diagnostic method for detecting hepatic steatosis and offers high accuracy in identifying moderate to severe fatty liver. However, its sensitivity is limited for mild cases and is highly dependent on the operator’s expertise and interpretation (7). Liver biopsy remains the gold standard for diagnosing MAFLD, as it allows for direct histological assessment of hepatic pathology and severity. Nevertheless, due to its invasive nature, high cost, and low feasibility in routine screening, especially among older adults with multiple comorbidities, its clinical applicability is limited (8, 9). In addition, many MAFLD patients—especially the elderly—may remain asymptomatic in the early stages, making timely and accurate diagnosis particularly challenging.

Machine learning (ML) has emerged as a powerful predictive tool in the field of medicine (10–12). Unlike traditional statistical models, which rely on predefined assumptions and explicit mathematical formulations, ML makes no assumptions about the underlying data structure. It is capable of analyzing high-dimensional data and capturing complex nonlinear relationships. Furthermore, the use of SHapley Additive exPlanations (SHAP) enhances the interpretability of ML models by combining optimal credit allocation with local interpretability (13). As a result, ML is increasingly applied in clinical diagnostic research.

This study aims to develop and validate machine learning models to predict the risk of MAFLD among older adults, utilizing SHAP to visualize and interpret key predictors. The goal is to assist clinicians in identifying high-risk individuals and supporting early clinical interventions.

## Methods

### Participants

This cross-sectional study was conducted between January 2024 and December 2024 at the Health Management Center of the Affiliated Hospital of Southwest Medical University. The study population comprised older adults who underwent annual health examinations, including abdominal ultrasonography. Inclusion criteria were as follows: (1) age ≥ 60 years; (2) completion of abdominal ultrasound examination; and (3) availability of complete clinical data. Exclusion criteria included: (1) age < 60 years; (2) a confirmed history of liver diseases or previous liver surgery, such as primary hepatocellular carcinoma, large hepatic cysts, or cirrhosis; and (3) incomplete clinical data. Based on these criteria, a total of 3,175 individuals with complete abdominal ultrasound data were initially assessed. After excluding 383 cases with missing data and 157 cases with major liver diseases, 2,635 participants were included in the final analysis. Among them, 1,693 were male (64.25%) and 942 were female (35.75%), with a mean age of 67.79 ± 7.07 years. Of the total participants, 878 (33.32%) were diagnosed with MAFLD and 1,757 (66.68%) were non-MAFLD. The diagnosis of MAFLD was based on ultrasonographic findings consistent with hepatic steatosis. All procedures complied with relevant ethical regulations and guidelines. All procedures in this study were conducted in accordance with the relevant guidelines and regulations. Due to the retrospective nature of the study, the requirement for written informed consent was waived. The study was approved by the Ethics Committee of the Affiliated Hospital of Southwest Medical University (Approval No. KY2025195).

### Data collection

Demographic, anthropometric, medical history, and laboratory data were extracted from the hospital’s electronic medical examination system. The collected variables included: Demographic Data: Age and sex. Anthropometric Measurements: Body mass index (BMI), systolic blood pressure (SBP), diastolic blood pressure (DBP), waist circumference (WC), hip circumference (HC), waist-to-hip ratio (WHR), height, and weight. Medical History: History of diabetes and history of hypertension (self-reported or clinically documented). Laboratory Tests: *γ*-glutamyl transpeptidase (GGT), alanine aminotransferase (ALT), aspartate aminotransferase (AST), AST/ALT ratio, low-density lipoprotein cholesterol (LDL-C), high-density lipoprotein cholesterol (HDL-C), total cholesterol (TC), total bilirubin (TBIL), direct bilirubin (DBIL), indirect bilirubin (IBIL), total protein (TP), globulin (GLO), triglycerides (TG), albumin (ALB), albumin-to-globulin ratio (A/G), and fasting plasma glucose (FPG). In addition, the triglyceride-glucose index (TyG) and its related parameters were calculated using the following formulas (14, 15):

TyG index = ln [TG (mg/dL) × FPG (mg/dL)/2].

TyG-BMI = TyG × BMI.

TyG-WC = TyG × WC.

TyG-WHR = TyG × WHR.

### Diagnostic criteria for MAFLD

In this study, all enrolled participants underwent abdominal ultrasonography performed by experienced radiologists at a tertiary medical center. The diagnosis of hepatic steatosis was primarily based on the following sonographic features: increased hepatic echogenicity (“bright liver”) and/or unclear visualization of intrahepatic structures (16). The diagnosis of metabolic dysfunction-associated fatty liver disease (MAFLD) was established based on the presence of hepatic steatosis on ultrasound in addition to at least one of the following three criteria (17): Overweight or obesity (defined as BMI ≥ 23 kg/m^2^ for Asian populations); Type 2 diabetes mellitus; Lean or normal weight (BMI < 23 kg/m^2^ for Asian populations) with the presence of two or more of the following metabolic risk abnormalities: (1) Waist circumference (WC) ≥ 90 cm in men or ≥ 80 cm in women; (2) Blood pressure ≥ 130/85 mmHg or under antihypertensive treatment; (3) Triglycerides (TG) ≥ 1.70 mmol/L or receiving lipid-lowering therapy; (4) HDL-C < 1.0 mmol/L in men or < 1.3 mmol/L in women, or receiving specific treatment; (5) Prediabetes (FPG 5.6–6.9 mmol/L or HbA1c 5.7–6.4%); (6) Homeostasis Model Assessment of Insulin Resistance (HOMA-IR) ≥ 2.5; (7) High-sensitivity C-reactive protein (hs-CRP) ≥ 2 mg/L.

### Statistical analysis and model development

All statistical analyses were conducted using R software (version 4.4.2), with a two-tailed *p*-value < 0.05 considered statistically significant. Continuous variables were expressed as mean ± standard deviation if normally distributed, or as median (interquartile range) if not. Group comparisons were performed using the *t*-test for normally distributed variables and the Mann–Whitney *U* test for non-normally distributed variables. Categorical variables were presented as frequencies (percentages) and compared using the chi-square test or Fisher’s exact test, as appropriate. We examined the missing rates of all variables included in the study. To ensure the accuracy and stability of the model, variables with a missing rate exceeding 10% were excluded from the analysis, while missing data for the remaining variables were imputed using the Multiple Imputation by Chained Equations (MICE) method.

**Figure 1:**
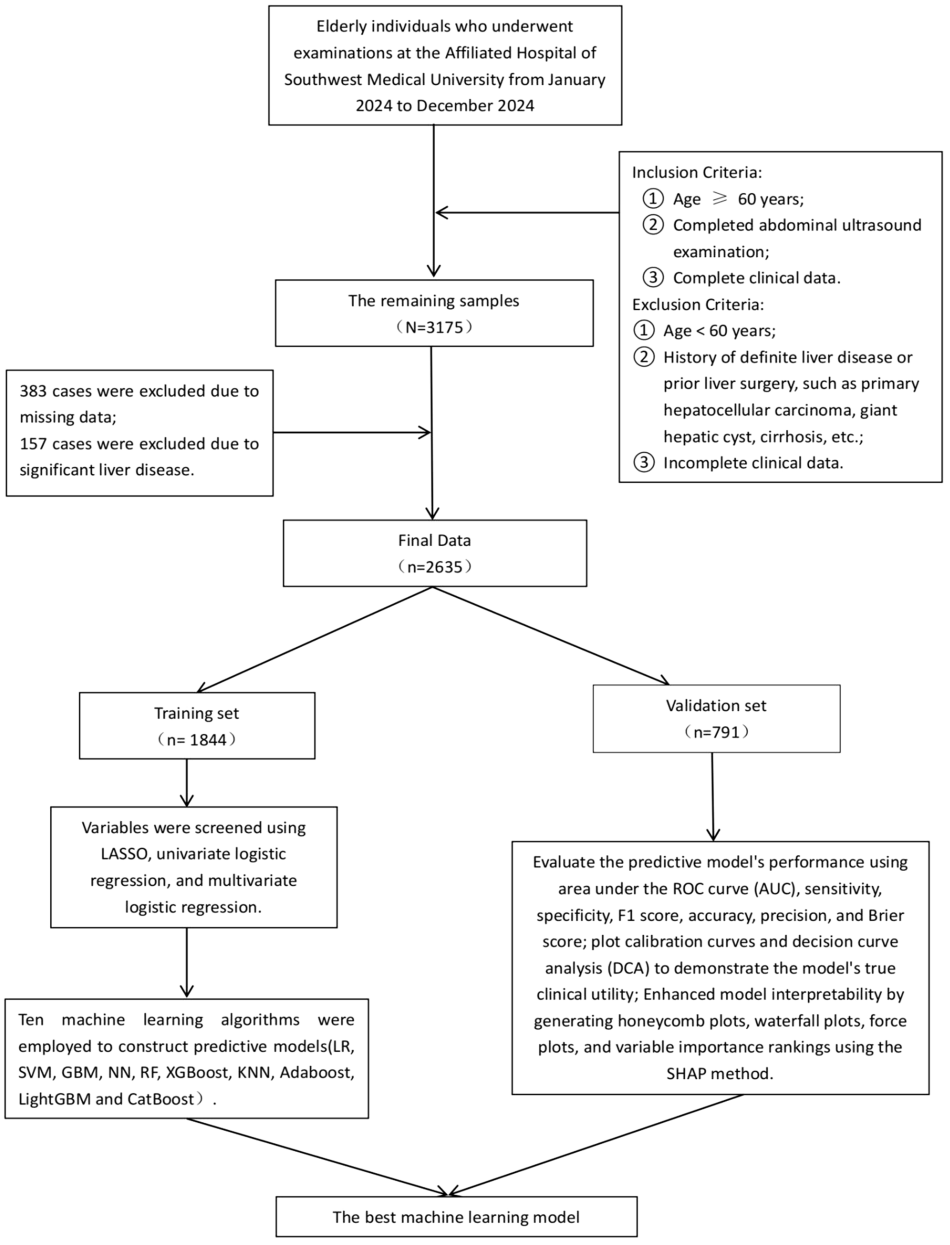
Research flowchart.

In this study, we used a stratified random sampling method to divide the dataset into a training set and a validation set. All participants were first stratified according to their MAFLD status, and then randomly assigned within each stratum to either the training set (70%) or the validation set (30%). The training set consisted of 1,844 individuals, and the validation set included 791 individuals. The training set was used for model development, while the validation set was used to evaluate model performance. Comparability between the two datasets was assessed, and no statistically significant differences were observed (*p* > 0.05). Variable selection was initially performed using least absolute shrinkage and selection operator (LASSO) regression on the training set. LASSO regression was implemented with the glmnet package in R, incorporating L1 regularization to penalize model complexity by shrinking some coefficients to zero, thereby achieving feature selection. The issue of class imbalance was addressed by introducing the Synthetic Minority Over-sampling Technique (SMOTE) algorithm (18). Subsequently, variables were further filtered through univariate logistic regression followed by multivariate logistic regression, resulting in the identification of nine independent predictors. The variance inflation factor (VIF) was calculated for each variable, and all VIF values were below 5, indicating no significant multicollinearity. To further eliminate the impact of multicollinearity on variable selection, we calculated the Pearson correlation coefficient between TyG-BMI and BMI, which was found to be 0.842. According to relevant literature (19–21), when the Pearson correlation coefficient exceeds 0.85, it is necessary to exclude one of the variables that has a weaker association with the outcome. Therefore, after comprehensive consideration, this study retains both TyG-BMI and BMI. The flowchart of this study is shown in Figure 1.

Based on a comprehensive consideration of methodological diversity, predictive performance, and clinical interpretability. Ten machine learning algorithms were employed to construct predictive models, including logistic regression (LR), support vector machine (SVM), gradient boosting machine (GBM), neural network (NN), random forest (RF), extreme gradient boosting (XGBoost), k-nearest neighbor (KNN), adaptive boosting (AdaBoost), light gradient boosting machine (LightGBM), and categorical boosting (CatBoost). Ten-fold cross-validation was used to ensure model robustness, and grid search was applied to optimize the hyperparameters of each algorithm.

### Model evaluation and interpretability

During hyperparameter tuning, the model with the highest area under the receiver operating characteristic (ROC) curve (AUC) was selected as the optimal model. The model was developed using the training set and internally validated using the optimal model. Model performance was evaluated based on AUC, sensitivity, specificity, F1-score, accuracy, precision, and Brier score. Additionally, calibration curves and decision curve analysis (DCA) were plotted to assess the model’s calibration and to demonstrate its potential clinical utility. To enhance model interpretability, SHapley Additive exPlanations (SHAP) were used to generate summary plots, waterfall plots, force plots, and feature importance rankings. This approach quantitatively illustrates the contribution of each feature to the model’s predictions (22, 23), thereby improving transparency and offering insight into how individual variables influence the model output.

## Results

### Baseline characteristics

All older adults were randomly divided into a training set (*n* = 1844, 70%) and a validation set (*n* = 791, 30%). Except for the variable hip circumference, no statistically significant differences were observed in baseline characteristics between the two groups (*p* > 0.05), indicating a balanced distribution of covariates (Table 1 and see Supplementary Material 1 for detailed information). Among the participants in the training set, 619 were diagnosed with MAFLD, yielding a prevalence rate of 33.57%. Significant differences in baseline characteristics were found between the MAFLD and non-MAFLD groups. Older adults with MAFLD exhibited notably abnormal metabolic indicators, including elevated levels of blood glucose, blood lipids, BMI, and liver function markers. Moreover, the prevalence of hypertension and diabetes was significantly higher in the MAFLD group compared to the non-MAFLD group (Table 2).

**Table 1:** Comparison of baseline data between training set and validation set.

Variables	Training set (*n* = 1844)	Validation set (*n* = 791)	Statistic	*P*
Age, Mean ± SD	67.81 ± 6.99	67.73 ± 7.24	*t* = −0.27	0.785
AST/ALT, Mean ± SD	1.24 ± 0.44	1.22 ± 0.43	*t* = −1.07	0.286
LDL-C, Mean ± SD	3.16 ± 0.90	3.09 ± 0.85	*t* = −1.88	0.060
TC, Mean ± SD	5.11 ± 1.14	5.05 ± 1.08	*t* = −1.25	0.211
TBIL, Mean ± SD	14.47 ± 5.30	14.71 ± 5.10	*t* = 1.07	0.286
TP, Mean ± SD	74.00 ± 4.52	73.66 ± 4.12	*t* = −1.89	0.058
GLO, Mean ± SD	30.08 ± 4.19	29.76 ± 3.77	*t* = −1.90	0.057
ALB, Mean ± SD	43.91 ± 2.63	43.90 ± 2.49	*t* = −0.11	0.909
A/G, Mean ± SD	1.49 ± 0.24	1.50 ± 0.23	*t* = 1.26	0.206
DBIL, Mean ± SD	3.15 ± 1.69	3.15 ± 1.65	*t* = 0.10	0.918
FPG, Mean ± SD	5.96 ± 1.81	5.98 ± 1.96	*t* = 0.23	0.819
IBIL, Mean ± SD	11.33 ± 4.28	11.55 ± 4.10	*t* = 1.25	0.211
HDL-C, Mean ± SD	1.48 ± 0.37	1.48 ± 0.38	*t* = 0.23	0.814
TyG, Mean ± SD	5.84 ± 0.62	5.84 ± 0.63	*t* = −0.10	0.918
TyG-WC, Mean ± SD	500.29 ± 85.88	502.64 ± 85.39	*t* = 0.65	0.519
TyG-BMI, Mean ± SD	141.06 ± 26.86	142.46 ± 26.27	*t* = 1.23	0.218
TyG-WHR, Mean ± SD	5.27 ± 0.75	5.27 ± 0.75	*t* = −0.11	0.914
WHR, Mean ± SD	0.90 ± 0.06	0.90 ± 0.06	*t* = 0.09	0.932
BMI, Mean ± SD	24.05 ± 3.18	24.31 ± 3.01	*t* = 1.94	0.053
Weight, Mean ± SD	61.87 ± 10.21	62.49 ± 10.02	*t* = 1.45	0.146
SBP, Mean ± SD	132.36 ± 17.47	132.64 ± 16.95	*t* = 0.38	0.706
DBP, Mean ± SD	74.24 ± 10.54	74.77 ± 10.34	*t* = 1.19	0.233
WC, Mean ± SD	85.35 ± 9.21	85.81 ± 8.92	*t* = 1.18	0.239
HC, Mean ± SD	94.67 ± 6.14	95.18 ± 5.84	*t* = 1.98	0.047
Height, Mean ± SD	162.10 ± 8.11	161.43 ± 8.48	*t* = −1.91	0.056
GGT, M (Q₁, Q₃)	23.10 (17.00, 33.50)	22.60 (16.85, 33.00)	*Z* = −0.32	0.752
ALT, M (Q₁, Q₃)	18.90 (14.70, 25.10)	19.40 (15.10, 25.95)	*Z* = −1.65	0.100
AST, M (Q₁, Q₃)	22.50 (19.38, 26.20)	22.80 (19.40, 26.90)	*Z* = −1.31	0.192
TG, M (Q₁, Q₃)	1.29 (0.95, 1.83)	1.28 (0.94, 1.82)	*Z* = −0.14	0.885
Result, *n*(%)			*χ*^2^ = 0.17	0.681
Non-MAFLD	1,225 (66.43)	532 (67.26)		
MAFLD	619 (33.57)	259 (32.74)		
Sex, *n*(%)			*χ*^2^ = 0.67	0.413
Male	1,194 (64.75)	499 (63.08)		
Female	650 (35.25)	292 (36.92)		
Hypertension, *n*(%)			*χ*^2^ = 3.74	0.053
No	929 (50.38)	431 (54.49)		
Yes	915 (49.62)	360 (45.51)		
Diabetes, *n*(%)			*χ*^2^ = 0.27	0.600
No	1,516 (82.21)	657 (83.06)		
Yes	328 (17.79)	134 (16.94)		

*t*, *t*-test; Z, Mann–Whitney test; *χ*^2^, Chi-square test; SD, standard deviation; M, median; Q₁, 1st Quartile; Q₃, 3st Quartile; BMI, Body Mass Index; SBP, systolic blood pressure; DBP, diastolic blood pressure; WC, waist circumference; HC, hip circumference; WHR, waist-to-hip ratio; GGT, gamma-glutamyl transferase; ALT, alanine aminotransferase; AST, aspartate aminotransferase; LDL-C, low-density lipoprotein cholesterol; HDL-C, high-density lipoprotein cholesterol; TC, total cholesterol; TBIL, total bilirubin; DBIL, direct bilirubin; IBIL, indirect bilirubin; TP, total protein; GLO, globulin; TG, triglycerides; ALB, albumin; A/G, albumin-to-globulin ratio; FPG, fasting plasma glucose.

**Table 2:** Distribution of baseline data in the training set.

Variables	Total data (*n* = 1844)	Non-MAFLD (*n* = 1,225)	MAFLD (*n* = 619)	Statistic	*P*
Age, Mean ± SD	67.81 ± 6.99	68.09 ± 7.22	67.25 ± 6.49	*t* = 2.52	0.012
AST/ALT, Mean ± SD	1.24 ± 0.44	1.32 ± 0.45	1.07 ± 0.35	*t* = 13.05	<0.001
LDL-C, Mean ± SD	3.16 ± 0.90	3.15 ± 0.91	3.18 ± 0.87	*t* = −0.74	0.461
TC, Mean ± SD	5.11 ± 1.14	5.11 ± 1.16	5.10 ± 1.10	*t* = 0.11	0.913
TBIL, Mean ± SD	14.47 ± 5.30	14.26 ± 5.07	14.89 ± 5.70	*t* = −2.43	0.015
TP, Mean ± SD	74.00 ± 4.52	73.79 ± 4.60	74.41 ± 4.34	*t* = −2.82	0.005
GLO, Mean ± SD	30.08 ± 4.19	30.36 ± 4.22	29.54 ± 4.10	*t* = 4.01	<0.001
ALB, Mean ± SD	43.91 ± 2.63	43.42 ± 2.51	44.88 ± 2.61	*t* = −11.59	<0.001
A/G, Mean ± SD	1.49 ± 0.24	1.46 ± 0.22	1.55 ± 0.25	*t* = −7.76	<0.001
DBIL, Mean ± SD	3.15 ± 1.69	3.11 ± 1.70	3.23 ± 1.67	*t* = −1.49	0.137
FPG, Mean ± SD	5.96 ± 1.81	5.72 ± 1.64	6.43 ± 2.04	*t* = −7.52	<0.001
IBIL, Mean ± SD	11.33 ± 4.28	11.16 ± 4.04	11.67 ± 4.69	*t* = −2.43	0.015
HDL-C, Mean ± SD	1.48 ± 0.37	1.57 ± 0.38	1.31 ± 0.29	*t* = 16.72	<0.001
TyG, Mean ± SD	5.84 ± 0.62	5.65 ± 0.53	6.23 ± 0.60	*t* = −20.43	<0.001
TyG-WC, Mean ± SD	500.29 ± 85.88	465.83 ± 69.10	568.48 ± 74.37	*t* = −29.35	<0.001
TyG-BMI, Mean ± SD	141.06 ± 26.86	129.51 ± 20.72	163.93 ± 22.64	*t* = −31.71	<0.001
TyG-WHR, Mean ± SD	5.27 ± 0.75	5.00 ± 0.63	5.81 ± 0.69	*t* = −25.24	<0.001
WHR, Mean ± SD	0.90 ± 0.06	0.88 ± 0.06	0.93 ± 0.06	*t* = −15.89	<0.001
BMI, Mean ± SD	24.05 ± 3.18	22.90 ± 2.68	26.34 ± 2.83	*t* = −25.56	<0.001
Weight, Mean ± SD	61.87 ± 10.21	58.80 ± 9.04	67.94 ± 9.66	*t* = −20.05	<0.001
SBP, Mean ± SD	132.36 ± 17.47	130.68 ± 17.33	135.68 ± 17.27	*t* = −5.86	<0.001
DBP, Mean ± SD	74.24 ± 10.54	73.60 ± 10.42	75.51 ± 10.68	*t* = −3.70	<0.001
WC, Mean ± SD	85.35 ± 9.21	82.36 ± 8.22	91.28 ± 8.13	*t* = −22.06	<0.001
HC, Mean ± SD	94.67 ± 6.14	93.03 ± 5.46	97.92 ± 6.10	*t* = −17.45	<0.001
Height, Mean ± SD	162.10 ± 8.11	160.01 ± 7.50	166.23 ± 7.69	*t* = −16.68	<0.001
GGT, M (Q₁, Q₃)	23.10 (17.00, 33.50)	21.00 (16.10, 29.90)	27.40 (19.70, 40.05)	*Z* = −9.07	<0.001
ALT, M (Q₁, Q₃)	18.90 (14.70, 25.10)	17.50 (13.70, 22.40)	22.60 (17.60, 29.40)	*Z* = −12.34	<0.001
AST, M (Q₁, Q₃)	22.50 (19.38, 26.20)	22.20 (19.10, 25.80)	22.70 (19.75, 27.40)	*Z* = −2.90	0.004
TG, M (Q₁, Q₃)	1.29 (0.95, 1.83)	1.13 (0.85, 1.49)	1.78 (1.29, 2.47)	*Z* = −18.37	<0.001
Sex, *n*(%)				*χ*^2^ = 0.49	0.482
Male	1,194 (64.75)	800 (65.31)	394 (63.65)		
Female	650 (35.25)	425 (34.69)	225 (36.35)		
Hypertension, *n*(%)				*χ*^2^ = 48.83	<0.001
No	929 (50.38)	688 (56.16)	241 (38.93)		
Yes	915 (49.62)	537 (43.84)	378 (61.07)		
Diabetes, *n*(%)				*χ*^2^ = 67.89	<0.001
No	1,516 (82.21)	1,071 (87.43)	445 (71.89)		
Yes	328 (17.79)	154 (12.57)	174 (28.11)		

*t*, *t*-test; Z, Mann–Whitney test; *χ*^2^, Chi-square test; SD, standard deviation; M, median; Q₁, 1st Quartile; Q₃, 3st Quartile; BMI, Body Mass Index; SBP, systolic blood pressure; DBP, diastolic blood pressure; WC, waist circumference; HC, hip circumference; WHR, waist-to-hip ratio; GGT, gamma-glutamyl transferase; ALT, alanine aminotransferase; AST, aspartate aminotransferase; LDL-C, low-density lipoprotein cholesterol; HDL-C, high-density lipoprotein cholesterol; TC, total cholesterol; TBIL, total bilirubin; DBIL, direct bilirubin; IBIL, indirect bilirubin; TP, total protein; GLO, globulin; TG, triglycerides; ALB, albumin; A/G, albumin-to-globulin ratio; FPG, fasting plasma glucose.

### Predictor selection

**Figure 2:**
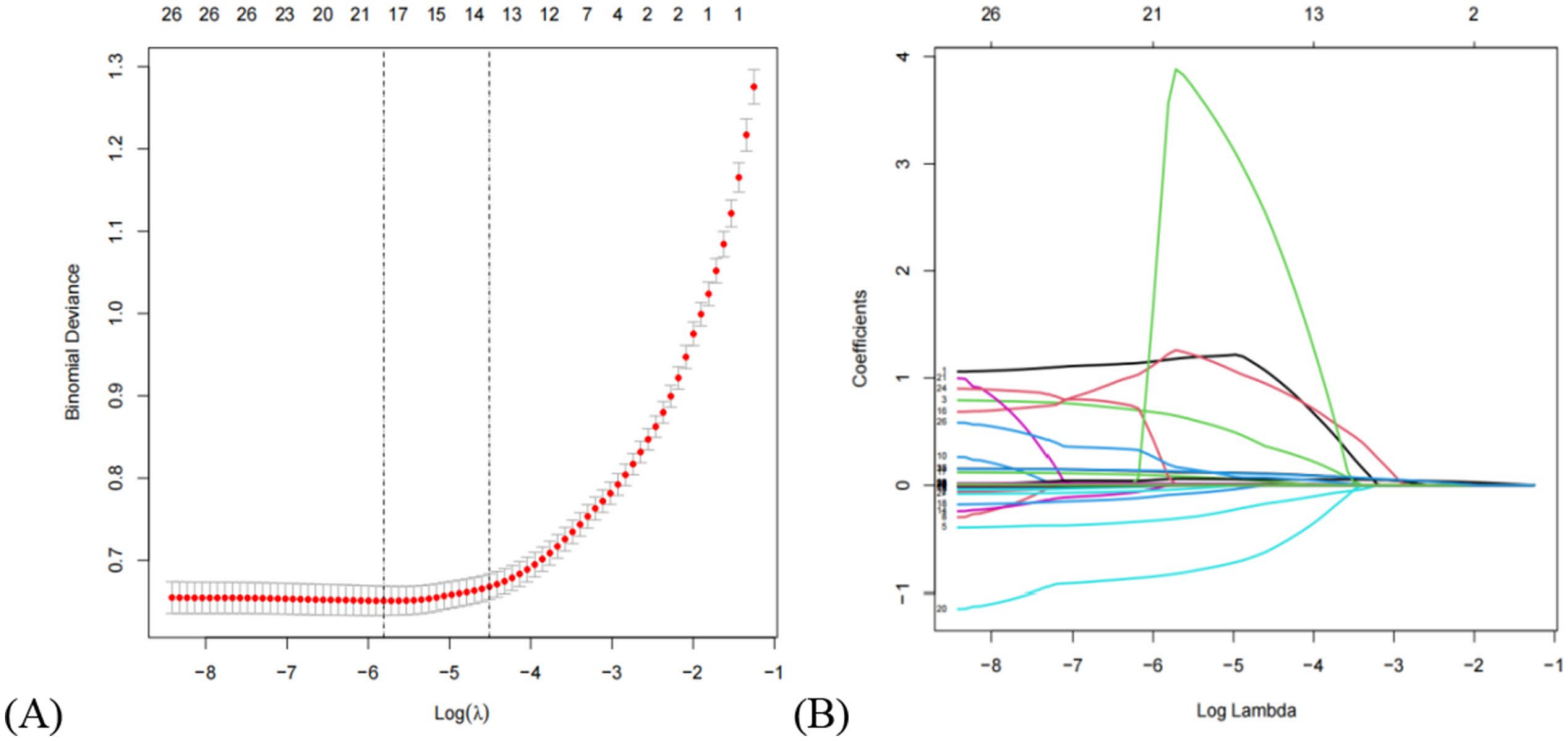
Clinical feature selection via the lasso regression model. **(A)** The partial likelihood deviance (binomial deviance) curve was plotted vs. log (lambda). The dotted vertical lines represent the optimal predictors using the minimum criteria (min. criteria) and the 1 SE of the minimum criteria (1-SE criteria). **(B)** Lasso coefficients of a total of 13 clinical features. Dynamic process diagram of lasso screening variables.

Based on cross-validation of the least absolute shrinkage and selection operator (LASSO) regression, two regularization parameters (*λ*) were determined: λ.min (0.002995174) and λ.1se (0.01101739). To achieve an optimal balance between model complexity and predictive accuracy, λ.1se (0.01101739)—which corresponded to the minimum cross-validation error—was selected as the optimal parameter. A total of 13 predictors were initially selected in the training set: sex, diabetes, AST/ALT, ALT, ALB, A/G, DBIL, HDL-C, TyG-BMI, WHR, BMI, SBP, and height. The LASSO selection process is illustrated in Figure 2. Subsequently, univariate and multivariate logistic regression analyses were performed to further refine the variable selection, and 9 independent predictors were ultimately identified: diabetes, ALT, ALB, A/G, HDL-C, TyG-BMI, BMI, SBP, and height (Table 3). Variance inflation factor (VIF) values were calculated for all variables, with all values below 5, indicating the absence of multicollinearity among predictors.

**Table 3:** Univariate and multivariate regression analysis of the variables after LASSO screening.

Variables	Univariate regression		Multivariate regression	
	OR (95%CI)	*P*	OR (95%CI)	*P*
Sex				
Male	1.00 (Reference)			
Female	1.07 (0.88 ~ 1.32)	0.482		
Diabetes				
No	1.00 (Reference)		1.00 (Reference)	
Yes	2.72 (2.13 ~ 3.47)	<0.001	1.79 (1.27 ~ 2.54)	<0.001
AST/ALT	0.13 (0.09 ~ 0.18)	<0.001		
ALT	1.04 (1.03 ~ 1.04)	<0.001	1.02 (1.01 ~ 1.03)	<0.001
ALB	1.26 (1.21 ~ 1.32)	<0.001	1.16 (1.08 ~ 1.24)	<0.001
A/G	5.41 (3.52 ~ 8.32)	<0.001	3.15 (1.53 ~ 6.45)	0.002
DBIL	1.04 (0.99 ~ 1.10)	0.138		
HDL-C	0.09 (0.06 ~ 0.12)	<0.001	0.54 (0.33 ~ 0.89)	0.015
TyG-BMI	1.08 (1.07 ~ 1.09)	<0.001	1.06 (1.05 ~ 1.07)	<0.001
WHR	525551.49 (82496.05 ~ 3348092.04)	<0.001		
BMI	1.60 (1.52 ~ 1.68)	<0.001	1.12 (1.03 ~ 1.22)	0.007
SBP	1.02 (1.01 ~ 1.02)	<0.001	1.01 (1.01 ~ 1.02)	0.001
Height	1.12 (1.10 ~ 1.13)	<0.001	1.11 (1.09 ~ 1.14)	<0.001

BMI, Body Mass Index; SBP, systolic blood pressure; WHR, waist-to-hip ratio; ALT, alanine aminotransferase; HDL-C, high-density lipoprotein cholesterol; DBIL, direct bilirubin; TP, total protein; GLO, globulin; TG, triglycerides; ALB, albumin; A/G, albumin-to-globulin ratio.

### Model development and performance evaluation

**Figure 3:**
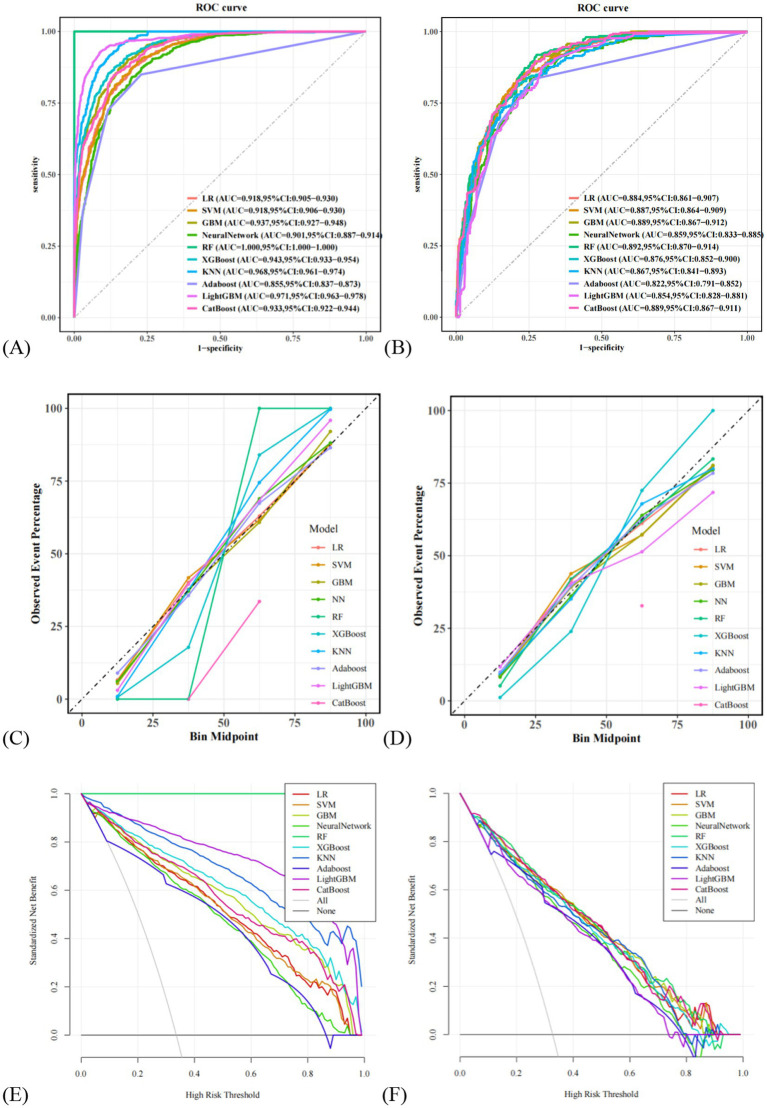
Comparison of the ROC curves for 10 machine learning models. **(A)** Comparison of ROC curves in the training set, **(B)** comparison of ROC curves on the validation set. **(C)** Comparison of calibration curves in the training set, **(D)** comparison of calibration curves on the validation set. **(E)** Comparison of DCA in the training set, **(F)** comparison of DCA on the validation set. LR, logistic regression; SVM, support vector machine; GBM, Gradient Boosting Machine; NN, NeuralNetwork; RF, random forest; XGBoost, eXtreme Gradient Boosting; KNN, K-Nearest Neighbor; Adaboost, Adaptive Boosting; LightGBM, Light Gradient Boosting Machine; CatBoost, Categorical Boosting.

In this study, 10 machine learning models were developed to assess the risk of MAFLD among older adults. A 10-fold cross-validation with grid search was applied to obtain the optimal hyperparameters for nine machine learning algorithms (excluding logistic regression, LR). Detailed information on the optimal hyperparameters for each model is available in Supplementary Material 1. Risk prediction models were subsequently constructed based on the optimal hyperparameters for each algorithm. The area under the receiver operating characteristic curve (AUC) was first used as the primary metric to evaluate model discrimination. In the validation set, the AUC values for each model were as follows: LR (0.884), SVM (0.887), GBM (0.889), NN (0.859), RF (0.892), XGBoost (0.876), KNN (0.867), Adaboost (0.822), LightGBM (0.854), and CatBoost (0.889). Among these, the random forest (RF) model demonstrated the best discriminatory performance. Further evaluation of model performance included accuracy, sensitivity, specificity, precision, F1 score, and Brier score. Detailed metrics for all 10 models are presented in Table 4. Notably, the RF model achieved the highest F1 score (0.739) and sensitivity (0.919), along with the lowest Brier score (0.125), indicating excellent predictive capability and calibration. Additionally, calibration curves and decision curve analysis (DCA) were plotted to assess the models’ calibration and clinical utility in both the training and validation sets (see ROC curves, calibration curves, and DCA in Figure 3). Taking all performance metrics into account, the RF model demonstrated the best overall performance, with strong calibration and clinical applicability, making it the most suitable predictive model in this study.

**Table 4:** Confusion matrix results of 10 machine learning models.

Data set	Model	Accuracy	Sensitivity	Specificity	Precision	F1 score	Brier score	AUC
Train	LR	0.828	0.871	0.806	0.694	0.772	0.110	0.918
	SVM	0.820	0.897	0.782	0.675	0.770	0.110	0.918
	GBM	0.844	0.903	0.815	0.711	0.796	0.095	0.937
	NN	0.815	0.842	0.802	0.682	0.753	0.122	0.901
	RF	1.000	1.000	1.000	1.000	1.000	0.015	1.000
	XGBoost	0.873	0.855	0.882	0.786	0.819	0.132	0.943
	KNN	0.882	0.948	0.848	0.759	0.843	0.077	0.968
	Adaboost	0.797	0.850	0.771	0.652	0.738	0.136	0.855
	LightGBM	0.917	0.929	0.911	0.841	0.883	0.059	0.971
	CatBoost	0.858	0.848	0.864	0.759	0.801	0.238	0.933
Valid	LR	0.803	0.842	0.784	0.655	0.736	0.131	0.884
	SVM	0.805	0.834	0.791	0.661	0.737	0.130	0.887
	GBM	0.785	0.888	0.735	0.620	0.730	0.129	0.889
	NN	0.775	0.834	0.746	0.615	0.708	0.143	0.859
	RF	0.788	0.919	0.724	0.618	0.739	0.125	0.892
	XGBoost	0.795	0.819	0.784	0.648	0.724	0.156	0.876
	KNN	0.804	0.734	0.838	0.688	0.710	0.138	0.867
	Adaboost	0.767	0.834	0.735	0.605	0.701	0.159	0.822
	LightGBM	0.761	0.873	0.707	0.592	0.705	0.160	0.854
	CatBoost	0.784	0.896	0.729	0.617	0.731	0.247	0.889

Train: training set; Valid, validation set; LR, logistic regression; SVM, support vector machine; GBM, Gradient Boosting Machine; NN, NeuralNetwork; RF, random forest; XGBoost, eXtreme Gradient Boosting; KNN, K-Nearest Neighbor; Adaboost, Adaptive Boosting; LightGBM, Light Gradient Boosting Machine; CatBoost, Categorical Boosting.

### Model interpretability

**Figure 4:**
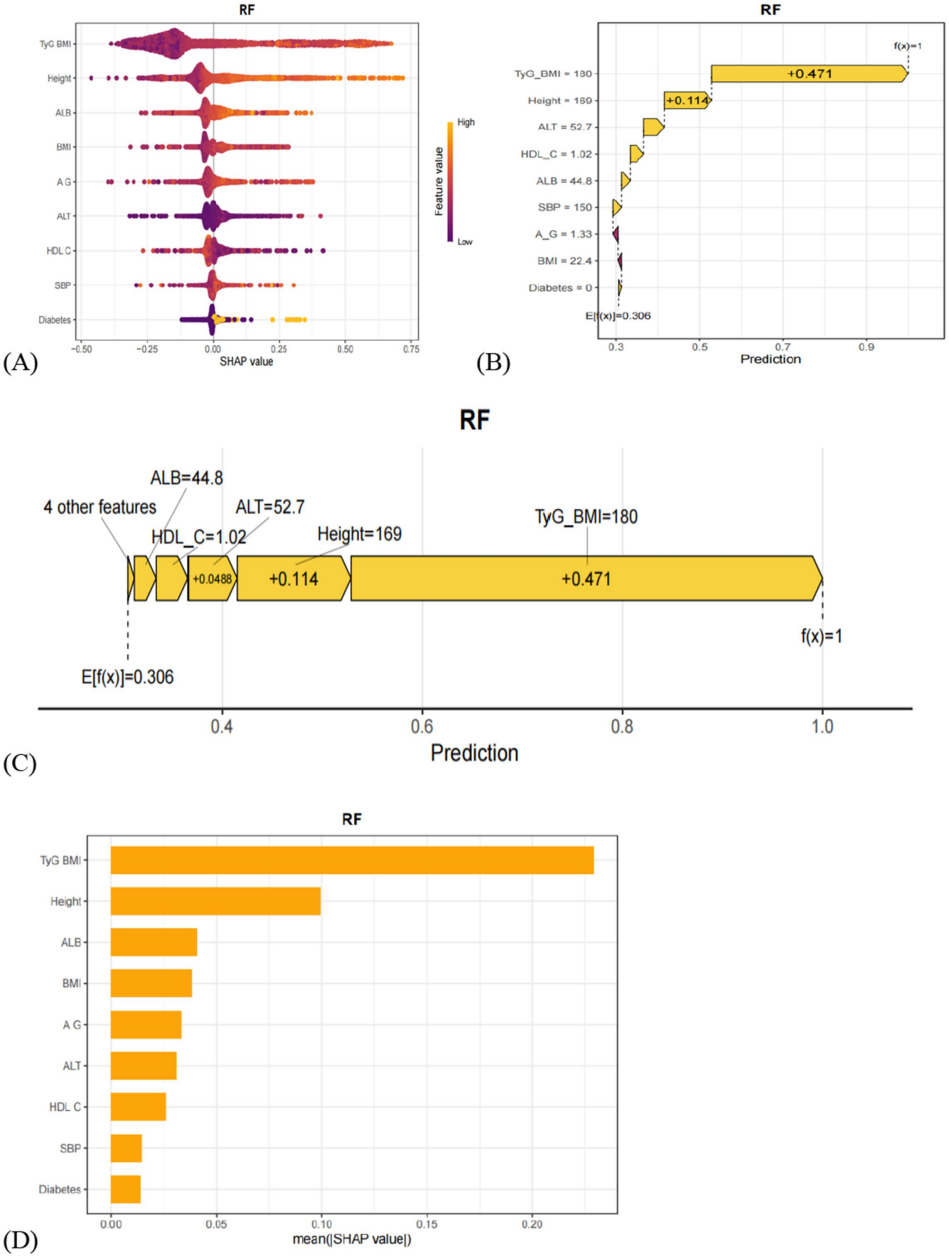
**(A)** Hive plot of the SHAP values of the model constructed by the RF algorithm. Vertical coordinates show the importance of the features, sorted in descending order of variable importance, while the variables above are more important to the model. For horizontal positions, the ‘Shap value’ shows whether the effect of this value is related to higher or lower predictions. The color of each SHAP value point indicates whether the observed value is high (yellow) or low (purple). **(B)** The waterfall plot of SHAP values for the model constructed by the RF algorithm. **(C)** SHAP value force plot of the model constructed using the RF algorithm. **(D)** The SHAP variable importance ranking plot of the model constructed using the RF algorithm.

To further interpret the results of the RF model, SHAP (SHapley Additive exPlanations) value-based visualizations were employed. As shown in Figure 4A, a summary (beeswarm) plot illustrates the distribution of SHAP values for each feature. In this plot, each point represents an individual patient; the *X*-axis indicates the magnitude and direction of the feature’s impact on the model output, while the *Y*-axis ranks the features by importance. Features positioned higher on the *Y*-axis have a greater influence on model predictions. The analysis identified nine key predictors for MAFLD in older adults: TyG-BMI, height, ALB, BMI, A/G, ALT, HDL-C, SBP, and diabetes. Among them, TyG-BMI, height, and ALB were the top three contributors to model predictions. Figures 4B,C present a detailed case study using SHAP waterfall and force plots to illustrate the prediction process for a specific individual. The waterfall plot reveals how the model prediction is formed by sequentially adding the SHAP values of individual features to a baseline value. The force plot offers a more intuitive visual summary of the collective “push and pull” effect of features on the prediction outcome for that patient. Additionally, Figure 4D displays a bar chart of feature importance ranked by their mean absolute SHAP values, clearly highlighting the relative contribution of each variable to the RF model. Features appearing at the top of the chart exert the most significant influence on the model’s predictions.

## Discussion

Metabolic-associated fatty liver disease (MAFLD) has a global prevalence of 38.77%, affecting more than one-third of the world’s population (24). A systematic review and meta-analysis forecast that by 2030, approximately 314.58 million people in China will be diagnosed with MAFLD (25). MAFLD has become an increasingly serious public health issue, imposing significant socioeconomic burdens. Epidemiological evidence indicates that the prevalence of MAFLD exhibits a distinct age-dependent pattern, with elderly individuals bearing a substantially higher burden of risk factors (26). Therefore, this study aims to develop machine learning models to enable early identification of high-risk elderly populations with MAFLD, thereby reducing medical and socioeconomic costs.

Our study identified TyG-BMI, height, albumin (ALB), body mass index (BMI), albumin/globulin ratio (A/G), alanine aminotransferase (ALT), systolic blood pressure (SBP), and diabetes as risk factors for MAFLD in the elderly, while high-density lipoprotein cholesterol (HDL-C) served as a protective factor. SHAP visualization further highlighted TyG-BMI, height, and ALB as the three most critical independent predictors.

TyG-BMI, a widely studied marker of metabolic dysregulation in recent years, integrates triglycerides (TG), fasting plasma glucose (FPG), and BMI, providing a comprehensive reflection of insulin resistance and metabolic abnormalities (27). Yang et al. (28) demonstrated a positive association between TyG-BMI and MAFLD, which remained significant after adjustments in multiple models. Additionally, a study based on the U. S. National Health and Nutrition Examination Survey (NHANES) data showed that TyG-BMI was significantly associated with all-cause mortality in MAFLD patients and had strong predictive value across different populations (29). Our findings that TyG-BMI is an independent predictor of MAFLD align with these previous reports.

Height emerged as a key predictor of MAFLD in our study, potentially related to differences in fat distribution among the elderly. Prior studies have shown significant correlations between height and both fat distribution and metabolic dysfunction, with taller individuals generally exhibiting higher basal metabolic rates and healthier fat distribution patterns (30–32). Albumin, synthesized by the liver (33), reflects hepatic synthetic function and reserve capacity. Chen et al. (34) reported that MAFLD patients tend to have lower ALB levels, indicating some degree of hepatic impairment. Li et al. (35) also found that decreased ALB levels were associated with an increased risk of MAFLD, potentially due to ALB’s anti-inflammatory and antioxidant properties. Our results corroborate these findings, confirming ALB as a risk factor for MAFLD in the elderly.

In addition to these three key predictors, BMI, A/G, ALT, SBP, and diabetes were also identified as risk factors for MAFLD in older adults. Studies have established a significant association between BMI and MAFLD risk, with BMI serving as a reliable predictor for MAFLD occurrence (36, 37). Due to hepatic fat accumulation and inflammation, immune activation leads to increased globulin synthesis, resulting in decreased A/G ratio. This change reflects hepatic synthetic function and overall health, indirectly indicating MAFLD risk (38). A prospective cohort study demonstrated that persistently high-normal ALT levels were significantly associated with increased risk of incident MAFLD, underscoring the importance of ALT monitoring for early identification of high-risk individuals (39). Furthermore, numerous studies have reported that MAFLD patients often present with hypertension and diabetes, with SBP ≥ 130 mmHg and diabetes significantly positively correlated with MAFLD risk (40–42).

HDL-C facilitates the transport of cholesterol from peripheral tissues to the liver for metabolism and excretion. One study indicated that low HDL-C levels may increase the risk of liver fibrosis and hepatocellular carcinoma in MAFLD patients, suggesting that higher HDL-C levels might be protective against MAFLD development, consistent with our findings (43).

Among the models developed, random forest (RF) demonstrated superior predictive accuracy and high sensitivity, making it the optimal model for predicting MAFLD risk in elderly populations. RF achieved the highest area under the ROC curve (AUC), with calibration curves closely aligned with the ideal line, and decision curve analysis (DCA) showing maximal net benefit across different threshold probabilities. At the same time, SHAP visualization was used to enhance the model’s interpretability, with the creation of hive plots, force plots, waterfall plots, and importance ranking plots for visual representation. These visualizations highlight how these factors interact and influence the prevalence of MAFLD in the elderly population. This interpretability ensures that the model is a transparent tool that clinicians and researchers can trust.

This study has several limitations. First, the RF model exhibited near-perfect performance on the training set, indicating a risk of overfitting. Although 10-fold cross-validation and regularization techniques were applied, further validation through nested cross-validation, early stopping, ensemble methods, or external validation on larger datasets is needed to ensure model robustness and generalizability. Second, all participants in this study were recruited from the Affiliated Hospital of Southwest Medical University, and the representativeness and regional applicability of the study population require further external validation using multi-center, large-scale clinical data to assess the generalizability of the findings. Third, this study is a cross-sectional study, and all sample data were drawn from the population undergoing health examinations at this hospital in 2024. Data from a single year may be subject to temporal and selection biases and cannot reflect the dynamic progression of the disease over time. Future studies should conduct prospective validation over longer follow-up periods and multiple time points to further ensure the robustness of the model. Fourth, the diagnosis of fatty liver disease in this study was based on abdominal ultrasound findings, which generally provide lower-level evidence compared to liver biopsy or magnetic resonance imaging (MRI). In addition, one of the diagnostic criteria for MAFLD is a plasma high-sensitivity C-reactive protein (hs-CRP) level ≥2 mg/L; however, this parameter was not routinely measured in the examined population. Other important factors affecting MAFLD risk, such as lifestyle habits and dietary patterns, were also not systematically recorded, which may have affected the accuracy of the prediction. Future research should aim to incorporate more comprehensive and detailed data to further enhance model performance and interpretability.

## Conclusion

The increasing prevalence of MAFLD among the elderly population has drawn considerable public attention, underscoring the need for large-scale early screening models tailored to this demographic. In this study, 10 machine learning models were developed and their performances compared, with the random forest model identified as the optimal predictor for MAFLD. Furthermore, SHAP visualization was employed to elucidate the interactions between various risk factors and MAFLD. The findings demonstrate that the proposed MAFLD screening model exhibits satisfactory predictive performance, offering a novel, cost-effective approach for the prevention and early detection of MAFLD in the elderly.

## Data Availability

Publicly available datasets were analyzed in this study. This data can be found here: Science Data Bank (ScienceDB), DOI: 10.57760/sciencedb.26204.
