# Supplementary material for: Predicting the risk of metabolic-associated fatty liver disease in the elderly population in China: construction and evaluation of interpretable machine learning models
Source: Front Med (Lausanne). 2025 Oct 20;12:1678076. doi: 10.3389/fmed.2025.1678076 (PMC12580202; doi:10.3389/fmed.2025.1678076)
Supplement: Supplementary file 1 [file Table_1.DOCX]

**Best parameters for each machine learning algorithm model**

| **Model** | **Parameters** |
| --- | --- |
| AdaBoost | mfinal=2, maxdepth=3 |
| CatBoost | tree_count=98, learning_rate=0.03, feature_count=7 |
| GBM | n.trees=100, interaction.depth=2, shrinkage=0.1, n.minobsinnode=5 |
| KNN | kmax=15, distance=1 |
| LightGBM | min_data=1, learning_rate =1, num_threads=2, verbosity=1, num_iterations=5, early_stopping_round=3 |
| NN | size=4, decay=0.6 |
| RF | mtry=3 |
| SVM | Sigma=0.01, C=0.5 |
| XGBoost | Nrounds=10, max_depth=5, eta=0.1, gamma=0.5, colsample_bytree=0.5, min_child_weight=1, subsample=0.6 |
